# Co-Ablation System for Pain Management in Bone Metastases: Retrospective Exploratory Study

**DOI:** 10.2196/86301

**Published:** 2026-02-19

**Authors:** Hang Yuan, Wei-Li Xia, Ho-Young Song, Lin Zheng, Wei-Jun Fan, Hong-Tao Hu

**Affiliations:** 1 The Affiliated Cancer Hospital of Zhengzhou University & Henan Cancer Hospital Zhengzhou, Henan China; 2 Sun Yat-sen University Cancer Center Guangzhou, Guangdong China

**Keywords:** bone metastases, pain management, co-ablation system, cryoablation, thermal ablation

## Abstract

**Background:**

Bone metastasis is a prevalent complication of malignant tumors, often resulting in restricted mobility, severe pain, and diminished quality of life.

**Objective:**

This study aimed to assess the analgesic effect and safety of a co-ablation system that combines elements of hyperthermic ablation and cryoablation in patients with bone metastases.

**Methods:**

This retrospective study included patients with histologically confirmed painful bone metastases treated with the co-ablation system between January and October 2024. Pain intensity was assessed using the numerical rating scale (NRS), and functional status was evaluated using the Karnofsky Performance Status score at baseline and during a 12-week follow-up. Technical success, adverse events, analgesic use, and pain response (defined as a ≥2-point reduction in NRS) were analyzed.

**Results:**

Nine patients were included. Technical success was achieved in all procedures, with no procedure-related adverse events observed. Median NRS scores decreased progressively from 5 (IQR 4-6) at baseline to 2 (IQR 1-3) at 12 weeks. By the fourth week, 8 (88.9%) patients achieved a clinically meaningful pain reduction. Karnofsky Performance Status scores showed gradual improvement during follow-up. No patients required escalation of analgesic therapy, and some experienced dose reductions.

**Conclusions:**

The co-ablation system appeared to be feasible and was associated with short-term pain reduction in patients with bone metastases, with no ablation-related adverse events observed.

## Introduction

Bone metastasis is a prevalent complication of malignant tumors, often resulting in restricted mobility, severe pain, and diminished quality of life [1]. Pain represents the most frequent symptom of bone metastasis [2]. Inadequate pain management is particularly prevalent among patients with bone metastases; approximately 80% of cancer-related pain originates from bone metastases, and as many as 25% of patients report insufficient symptom control [3]. In addition, chronic pain can cause depression and anxiety [4,5]. Currently, numerous treatment options exist for managing pain associated with bone metastases, including systemic therapies (chemotherapy, hormonal therapy, radiopharmaceuticals, and bisphosphonates), various analgesics, local interventions (radiotherapy, laser-induced interstitial thermotherapy, and ablation therapy), and surgical approaches [6-8].

Currently, the most prevalent form of local ablation used in analgesic treatment includes techniques such as radiofrequency ablation, cryoablation, and microwave ablation. These methods have been extensively validated for their safety and analgesic effect [9,10]. However, each modality has inherent limitations. Hyperthermic ablation techniques (radiofrequency ablation and microwave ablation) may be constrained by heat-sink effects and carry a risk of thermal injury to adjacent structures [11,12], whereas cryoablation, although allowing improved visualization of the ice ball, does not permit needle tract ablation.

Chinese scientists have developed a co-ablation system that combines elements of hyperthermic ablation and cryoablation, using liquid nitrogen and alcohol as thermal media. The system is capable of achieving temperatures as low as −196 °C in freezing mode and up to approximately 80 °C in heating mode. The system operates by sequential application of freezing and heating, delivering extreme temperature changes to the target tissue. This design is intended to provide controlled thermal injury through alternating cold and heat exposure. Previous studies have demonstrated its effectiveness in cancer treatment [13,14].

However, there is currently limited literature documenting the use of a co-ablation system for the analgesic treatment of bone metastases. To address this knowledge gap, we conducted a retrospective exploratory study to assess the effectiveness and safety of the co-ablation system in managing pain associated with bone metastases.

## Methods

### Ethical Considerations

This was a retrospective study approved by the ethics committee of Henan Cancer Hospital (2025-KY-0026). Informed consent was waived by the ethics committee. All patients signed the procedural informed consent form and the general informed consent for anonymous use of their information in research before receiving treatment. During statistical analysis, all patient information was anonymized. Therefore, this study does not involve informed consent, privacy, or compensation.

### Participants

From January to October 2024, we retrospectively analyzed patients with histologically confirmed bone metastases treated with the co-ablation system.

The exclusion criteria included the following: (1) survival time less than 3 months, (2) lack of a definitive pathological diagnosis, (3) the ablation site was not a bone metastasis, (4) absence of pain before ablation, and (5) loss to follow-up.

### Procedure

According to the tumor size, appropriate co-ablation needles (RBL20, RCS17, and RAL26; Hygea Medical Technology Co) were selected preoperatively, and their functionality was verified using normal saline. Patients were positioned in a supine, prone, or lateral position on the computed tomography (CT) examination bed (GE Large Aperture CT; GE Healthcare Bio-Sciences Corp). A conventional CT scan was initially performed to identify the optimal puncture pathway, which was then marked and subsequently sterilized. Under local anesthesia, the bone metastatic lesion was gradually punctured, and the correct positioning was confirmed before connecting the co-ablation system (Hygea Medical Technology Co) for ablation treatment. Relevant parameters, including freezing time and rewarming time, were adjusted according to the extent of the tumor. Immediate postablation CT imaging was performed to evaluate technical adequacy. The assessment included confirmation of complete coverage of the target lesion by the ablation zone; appropriate extension beyond the lesion margins when feasible; and the absence of procedure-related adverse events (AEs), such as hemorrhage, pathological fracture, or injury to adjacent critical structures.

### Data Collection and Follow-Up

Patient pain was assessed using a numerical rating scale (NRS) [15], and the Karnofsky Performance Status (KPS) score was used to evaluate patients’ physical condition. NRS scores were recorded before the procedure and at 1 day, 1 week, 4 weeks, 8 weeks, and 12 weeks after the procedure. Pain assessment primarily targeted pain related to the treated bone metastatic lesion. Patients were instructed to focus on pain originating from the target lesion identified for the co-ablation system. KPS scores were recorded before the procedure and 1, 4, 8, and 12 weeks after the procedure. For patients outside the hospital, follow-up assessments were conducted via telephone interviews.

Age, gender, primary tumor type, location and number of bone metastases, administration of bone-modifying agents, use of analgesic drugs, dose adjustments, procedure-related AEs, and other concomitant treatments were reviewed through the electronic medical record system. All procedure-related AEs were recorded and classified according to the Society of Interventional Radiology AE classification system [16]. AEs were graded as mild, moderate, severe, life-threatening, or fatal based on clinical severity, imaging findings, and the requirement for medical intervention or prolonged hospitalization. Technical success and pain response were assessed for each procedure.

Technical success was defined as successful placement of the needle into the target lesion and completion of the planned protocol without technical failure or premature termination. Clinical success was predefined as a clinically meaningful pain reduction, defined as a decrease of ≥2 points in the NRS score compared with baseline [17]. Patients meeting this criterion at a given follow-up time point were classified as responders, and the corresponding proportion was reported as the responder rate.

### Statistical Analysis

Statistical analyses were performed using GraphPad Prism software (version 9.5; GraphPad Software). Continuous and ordinal variables were presented as median and IQR. Changes in NRS and KPS scores across multiple time points were analyzed using the Friedman test. When the Friedman test indicated a statistically significant difference, post hoc comparisons between baseline and follow-up time points were performed using Dunn multiple comparisons test with adjustment for multiple testing. All tests were 2-sided, and an adjusted *P*<.05 was considered statistically significant.

## Results

### Participant Characteristics

**Figure 1:**
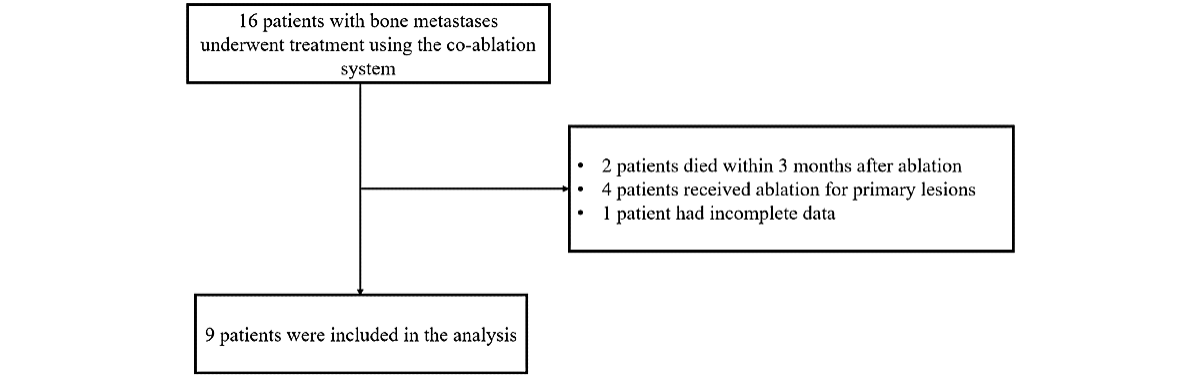
Flow diagram of patient enrollment and study inclusion. A total of 16 patients with bone metastases underwent treatment using the co-ablation system. Of these, 2 patients died within 3 months after ablation, 4 patients received ablation for primary lesions, and 1 patient had incomplete clinical data. The remaining 9 patients met the inclusion criteria and were included in the final analysis.

From January 2024 to October 2024, a total of 16 patients with bone metastases were treated with the co-ablation system, among whom 2 died within 3 months after treatment, 4 had primary lesions, and 1 was excluded due to loss of follow-up data. Finally, 9 patients were included in the analysis (Figure 1), including 6 male patients and 3 female patients, with a median age of 60 (IQR 55-69) years. Primary malignancies included hepatocellular carcinoma (n=4, 44%), lung adenocarcinoma (n=4, 44%), and colon adenocarcinoma (n=1, 11%). All patients had a single bone metastasis lesion. Metastatic involvement was primarily located in the femur (n=3, 33%), ilium (n=4, 44%), and ribs (n=2, 22%). Treatment regimens varied among patients: 1 (11%) patient received denosumab injections, 5 (56%) patients were treated with zoledronic acid, and 3 (33%) patients received ibandronate. All patients continued to undergo systemic antitumor therapy (Table 1).

**Table 1:** Patient characteristics (N=9).

Characteristic			Values
Age (years), median (IQR)			60 (55-69)
**Sex, n (%)**			
	Male	6 (67)	
	Female	3 (33)	
**Primary tumor type, n (%)**			
	Hepatocellular carcinoma	4 (44)	
	Lung adenocarcinoma	4 (44)	
	Colon adenocarcinoma	1 (11)	
**Metastatic site, n (%)**			
	Femur	3 (33)	
	Ilium	4 (44)	
	Rib	2 (22)	
	Weight-bearing lesion	7 (78)	
Baseline NRS^a^ score, median (IQR)			5 (4-6)
Baseline KPS^b^ score, median (IQR)			60 (50-70)
Baseline analgesic use, n (%)			9 (100)
**Use of bone-modifying agents, n (%)**			
	Denosumab	1 (11)	
	Zoledronic acid	5 (56)	
	Ibandronate	3 (33)	

^a^NRS: numerical rating scale.

^b^KPS: Karnofsky Performance Status.

### Pain Intensity Outcomes

Pain intensity assessed using the NRS showed a significant change over time (*P*<.001). An overall comparison using the Friedman test demonstrated a statistically significant difference in NRS scores across all assessed time points (baseline, 1 day, 1 week, 4 weeks, 8 weeks, and 12 weeks after treatment; *P*<.001).

Median NRS scores decreased from 5 (IQR 4-6) at baseline to 4 (IQR 3-5) at 1 day and 3 (IQR 2-4) at 1 week after treatment. Further reductions were observed at 4 weeks (median 2, IQR 2-3) and 8 weeks (median 2, IQR 1-2), with improvement maintained at 12 weeks (median 2, IQR 1-3; Table 2).

**Table 2:** Changes in the numerical rating scale and Karnofsky Performance Status scores over time.

Time point	Numerical rating scale, median (IQR)	Karnofsky Performance Status, median (IQR)
Baseline	5 (4-6)	60 (50-70)
1 day after treatment	4 (4-5)	—^a^
1 week after treatment	3 (2-4)	60 (50-80)
4 weeks after treatment	3 (2-3)	70 (60-80)
8 weeks after treatment	2 (1-2)	70 (70-90)
12 weeks after treatment	2 (2-3)	70 (60-90)

^a^Not available.

**Figure 2:**
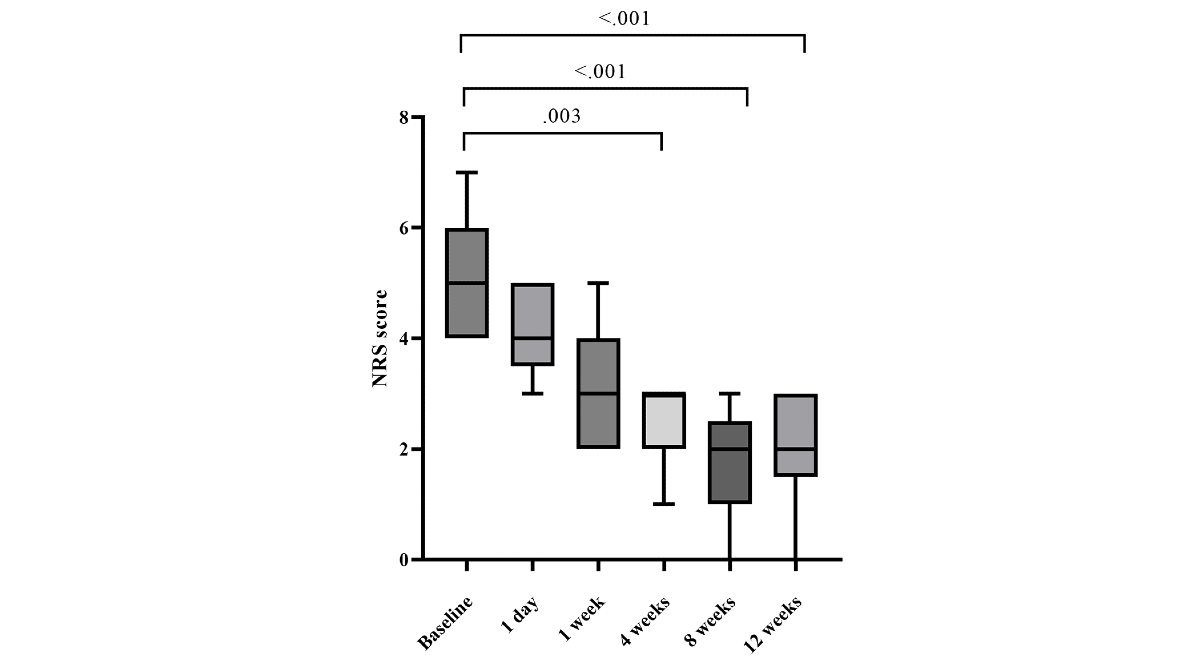
Changes in pain intensity (numerical rating scale [NRS] scores) over time after the procedure. Box-and-whisker plots show NRS scores at baseline and at 1 day, 1 week, 4 weeks, 8 weeks, and 12 weeks after treatment. Data are presented as median, IQR (box), and range (whiskers). Overall differences across time points were assessed using the Friedman test. Post hoc comparisons between baseline and follow-up time points were performed using the Dunn multiple comparisons test with adjustment for multiple testing. Adjusted *P* values for statistically significant comparisons are shown.

Compared with baseline, NRS scores were significantly lower at 1 week (adjusted *P*=.003), 8 weeks (adjusted *P*<.001), and 12 weeks (adjusted *P*<.001; Figure 2). By week 4, among the 9 patients, a clinically meaningful pain reduction (≥2-point decrease in NRS) was observed in 8.

### Functional Status Outcomes

Functional performance, as measured by the KPS score, improved over the course of follow-up. A significant difference in KPS scores was observed across baseline and subsequent assessment time points (*P*<.001).

At baseline, patients demonstrated limited functional status, with a median KPS score of 60 (IQR 50-70). An increase in KPS was observed at 1 week after treatment (median 60, IQR 50-80); however, this early change was not statistically significant (adjusted *P*=.54). In contrast, KPS scores showed significant improvement at later follow-up visits, reaching a median of 70 (IQR 60-80) at 4 weeks and 70 (IQR 70-90) at 8 weeks, with these improvements maintained through 12 weeks (median 70, IQR 60-90; Table 2).

**Figure 3:**
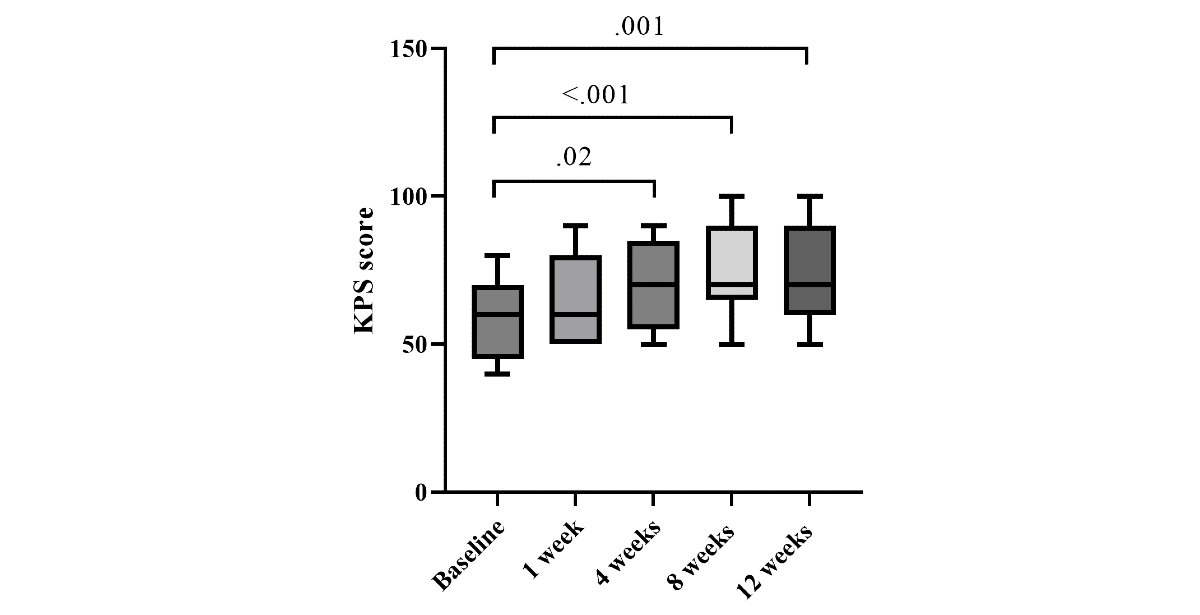
Changes in functional status (Karnofsky Performance Status [KPS] scores) during follow-up. Box-and-whisker plots illustrate KPS scores at baseline and at 1, 4, 8, and 12 weeks after co-ablation. Values are expressed as median, IQR (box), and range (whiskers). Longitudinal changes were analyzed using the Friedman test, followed by the Dunn post hoc test with correction for multiple comparisons. Adjusted *P* values for significant differences compared with baseline are indicated.

Compared with baseline, KPS scores were significantly higher at 4 weeks (adjusted *P*=.02), 8 weeks (adjusted *P*<.001), and 12 weeks (adjusted *P*=.001; Figure 3).

### AEs and Analgesic Dosage

All patients underwent successful treatment with the co-ablation system, achieving a technical success rate of 100% without any procedure-related AEs. At the 12-week follow-up, no patients switched analgesic agents, 3 patients experienced a 25% reduction in their analgesic dosage, 1 patient experienced a 50% reduction, and no patients required an increase in medication dosage.

## Discussion

In this retrospective single-arm exploratory study, the use of the co-ablation system was feasible and well tolerated in patients with bone metastases, with no procedure-related AEs observed. A reduction in pain intensity was noted over the follow-up period, with NRS scores showing a statistically significant overall decrease and most patients achieving a clinically meaningful pain reduction by week 4. Improvements in functional status, as reflected by KPS scores, were observed at later follow-up time points; however, these findings should be interpreted cautiously given the small sample size and study design. No patients required escalation of analgesic therapy during follow-up, and some were able to reduce their baseline analgesic doses. Collectively, these preliminary findings support the technical feasibility and short-term safety of the co-ablation system for palliative pain management in bone metastases.

Several studies have reported favorable pain control outcomes following ablation for bone metastases. Ma et al [18] evaluated microwave ablation, radiofrequency ablation, and cryoablation in patients with bone metastases from non**-**small cell lung cancer and observed a substantial reduction in pain scores at 4 weeks after treatment. Consistent with these findings, our study demonstrated a marked decrease in NRS scores over time, with median pain scores declining from baseline to 4 weeks after the procedure and most patients achieving a clinically meaningful pain reduction. Although an early reduction in pain was observed as soon as 1 day after treatment in our cohort, this change did not reach statistical significance, which may be related to the small sample size. In contrast, Yang et al [19] reported significant pain relief within 1 day following cryoablation; however, pain was assessed using a visual analog scale rather than NRS, which may limit direct comparability. Additionally, Tomasian et al [20] reported sustained pain relief at 3 months after ablation for painful vertebral metastases, a finding that aligns with the maintained reduction in NRS scores observed through 12 weeks in our study. Taken together, these observations suggest that the co-ablation system may offer pain control outcomes comparable to established ablation modalities, although direct comparisons should be interpreted cautiously.

High-intensity focused ultrasound is a noninvasive image-guided ablation technique that has demonstrated a favorable safety profile and promising analgesic effects in patients with painful bone metastases [21]. Previous prospective studies have shown that magnetic resonance–guided high-intensity focused ultrasound (MR-HIFU) can achieve rapid and clinically meaningful pain reduction without treatment-related AEs [22,23]. Bongiovanni et al [22] reported complete or partial pain responses in all treated patients at 30 days, whereas Napoli et al [23] observed significant improvements in pain severity and interference, with additional evidence of local tumor response in some cases. Although MR-HIFU differs from the co-ablation system evaluated in this study in terms of invasiveness and ablation mechanism, both approaches support the role of targeted local ablation in pain palliation. In our cohort, short-term pain relief without ablation-related AEs was observed, which is consistent with the analgesic and safety trends reported for MR-HIFU.

Previous studies have shown that multimodality approaches combining local ablation with radiotherapy, vertebroplasty, or iodine-125 seed implantation can improve pain control in patients with bone metastases [24-26]. These findings suggest that co-ablation could potentially be incorporated into multimodal palliative strategies in future practice and investigation. In our study, we also observed that the dosage of analgesic drugs was significantly reduced in some patients following the procedure. This finding is consistent with previous reports indicating a reduction in analgesic requirements after cryoablation [19,20,24,27].

Following treatment with the co-ablation system, patients demonstrated a gradual improvement in functional status, as reflected by increasing KPS scores over the follow-up period. Although no statistically significant improvement was observed at 1 week after treatment, KPS scores improved significantly from 4 weeks onward and remained stable through 12 weeks. These findings are in line with the MOTION (Multicenter Study of Cryoablation for Palliation of Painful Bone Metastases) study, which reported that cryoablation provided rapid pain relief while functional status was maintained and quality of life improved during follow-up [28].

This study is limited by its small sample size and retrospective, single-arm design; these factors reduce statistical power, limit generalizability, and preclude adjustment for pain-related confounders. Patient heterogeneity in primary tumors and metastatic sites, as well as the absence of a comparative control group, prevents assessment of relative efficacy vs established ablation modalities. The 12-week follow-up period was sufficient for short-term pain assessment but inadequate for evaluating the durability of response, pain recurrence, or delayed AEs. Pain from untreated disease sites may have influenced NRS and KPS scores, and the use of KPS without patient-reported outcome measures provides limited insight into overall quality of life.

In this small retrospective exploratory study, the co-ablation system was technically feasible and well tolerated in patients with painful bone metastases. Pain reduction was observed during short-term follow-up, and functional performance was maintained or gradually improved, with no procedure-related AEs recorded. These preliminary findings support further evaluation of the co-ablation system in larger, prospective controlled studies.

## Abbreviations

AE: adverse event
CT: computed tomography
KPS: Karnofsky Performance Status
MOTION: Multicenter Study of Cryoablation for Palliation of Painful Bone Metastases
MR-HIFU: magnetic resonance–guided high-intensity focused ultrasound
NRS: numerical rating scale

## Data Availability

The datasets generated or analyzed during this study are available from the corresponding author on reasonable request.
